# Translating HbA_1c_ measurements into estimated average glucose values in pregnant women with diabetes

**DOI:** 10.1007/s00125-017-4205-7

**Published:** 2017-01-19

**Authors:** Graham R. Law, Mark S. Gilthorpe, Anna L. Secher, Rosemary Temple, Rudolf Bilous, Elisabeth R. Mathiesen, Helen R. Murphy, Eleanor M. Scott

**Affiliations:** 1 School of Health and Social Care, University of Lincoln, Lincoln, UK; 2 Division of Epidemiology and Biostatistics, Leeds Institute of Cardiovascular and Metabolic Medicine, Clarendon Way, University of Leeds, Leeds, LS2 9JT UK; 3 Institute for Clinical Medicine, University of Copenhagen, Copenhagen, Denmark; 4 Elsie Bertram Diabetes Centre, Norfolk and Norwich University Hospital NHS Trust, Norwich, UK; 5 Newcastle University Medicine Malaysia, Johor, Malaysia; 6 Norwich Medical School, University of East Anglia, Norwich, UK

**Keywords:** Average glucose, Continuous glucose monitoring, Estimated average glucose, Gestation, HbA_1c_, Pregnant, Type 1 diabetes, Type 2 diabetes

## Abstract

**Aims/hypothesis:**

This study aimed to examine the relationship between average glucose levels, assessed by continuous glucose monitoring (CGM), and HbA_1c_ levels in pregnant women with diabetes to determine whether calculations of standard estimated average glucose (eAG) levels from HbA_1c_ measurements are applicable to pregnant women with diabetes.

**Methods:**

CGM data from 117 pregnant women (89 women with type 1 diabetes; 28 women with type 2 diabetes) were analysed. Average glucose levels were calculated from 5–7 day CGM profiles (mean 1275 glucose values per profile) and paired with a corresponding (±1 week) HbA_1c_ measure. In total, 688 average glucose–HbA_1c_ pairs were obtained across pregnancy (mean six pairs per participant). Average glucose level was used as the dependent variable in a regression model. Covariates were gestational week, study centre and HbA_1c_.

**Results:**

There was a strong association between HbA_1c_ and average glucose values in pregnancy (coefficient 0.67 [95% CI 0.57, 0.78]), i.e. a 1% (11 mmol/mol) difference in HbA_1c_ corresponded to a 0.67 mmol/l difference in average glucose. The random effects model that included gestational week as a curvilinear (quadratic) covariate fitted best, allowing calculation of a pregnancy-specific eAG (PeAG). This showed that an HbA_1c_ of 8.0% (64 mmol/mol) gave a PeAG of 7.4–7.7 mmol/l (depending on gestational week), compared with a standard eAG of 10.2 mmol/l. The PeAG associated with maintaining an HbA_1c_ level of 6.0% (42 mmol/mol) during pregnancy was between 6.4 and 6.7 mmol/l, depending on gestational week.

**Conclusions/interpretation:**

The HbA_1c_–average glucose relationship is altered by pregnancy. Routinely generated standard eAG values do not account for this difference between pregnant and non-pregnant individuals and, thus, should not be used during pregnancy. Instead, the PeAG values deduced in the current study are recommended for antenatal clinical care.

**Electronic supplementary material:**

The online version of this article (doi:10.1007/s00125-017-4205-7) contains peer-reviewed but unedited supplementary material, which is available to authorised users.

## Introduction

The relationship between HbA_1c_ and average glucose levels has been explored in many studies, most making use of intermittent capillary blood glucose measurements [1–6]. More recently, intensive longitudinal data from continuous glucose monitoring (CGM) have been used to derive a more accurate picture of how average glucose levels compare with HbA_1c_ over time [7–11]. The A1C-Derived Average Glucose (ADAG) study showed a linear association between CGM-measured average glucose and HbA_1c_ levels in non-pregnant adults with type 1 and type 2 diabetes [8]. Following endorsement of the ADAG analysis by the ADA, EASD, International Diabetes Federation (IDF) and International Federation of Clinical Chemists (IFCC) [12], many laboratories now report HbA_1c_ data as a standard estimated average glucose (eAG) alongside the HbA_1c_ result, facilitating greater patient understanding of how daily glucose measurements relate to HbA_1c_ levels.

The ability to accurately assess glucose control is critical in the context of pregnancy in women with diabetes, where achieving tight glucose control has a beneficial impact on maternal–fetal health outcomes. However, HbA_1c_ is considered unreliable for assessing glucose control during pregnancy owing to physiological changes that may be attributed to increased red cell production, shortened red cell life span, reduced red cell affinity for glucose, iron deficiency and iron supplementation [13–17]. This has led to uncertainty over the role of HbA_1c_ for blood glucose assessment in pregnancy [18], with key bodies [19, 20] advising that it should not be used for diagnosing diabetes in pregnancy, and the National Institute for Health and Care Excellence (NICE) in the UK recommending that it should not be routinely used to assess glucose control in pregnancy in women with established diabetes [20]. Furthermore, the relationship between any physiological changes in HbA_1c_ across pregnancy and average glucose levels obtained by CGM is unknown. Despite these limitations, HbA_1c_ is widely used in clinical practice during pregnancy in the UK [21], the USA [22] and internationally [23]. Anecdotal reports also suggest that clinicians and patients are using the standard eAG value, which is reported with HbA_1c_ levels, during pregnancy, despite it being derived from data from non-pregnant adults.

Thus, the aims of this analysis were to: (1) examine the relationship between average glucose levels assessed by CGM and HbA_1c_ levels in pregnancy in women with type 1 and type 2 diabetes; (2) determine if this relationship changes with gestational week during pregnancy; and (3) determine whether the standard eAG calculation that is derived from HbA_1c_ measurements is applicable to pregnant women with diabetes.

## Methods

### Participants

This analysis used data obtained from two previously published studies: one based in the UK (East Anglia) [24] and the second in Denmark (Copenhagen) [25]. Both studies recruited pregnant women with pregestational type 1 or type 2 diabetes to prospective randomised controlled trials that explored the clinical impact of CGM on maternal, fetal and neonatal health outcomes. In the UK, pregnant participants, aged 16–45 years, were recruited from two secondary care diabetes antenatal clinics between 2003 and 2006. In Denmark, pregnant participants, aged 19–43 years, were recruited from one diabetes antenatal clinic between 2009 and 2011. Full details of clinical recruitment procedures (including the exclusion of participants with severe medical or psychological comorbidities) have been described previously [24–26]. A total of 117 participants (49 from England and 68 from Denmark), comprising 89 women with type 1 diabetes and 28 with type 2 diabetes, were included in the present analysis [26].

All participants gave written informed consent. Ethical approval was granted by the Suffolk and Norfolk Local Research Ethics Committee and the Danish National Committee on Biomedical Research Ethics. The Helsinki Declaration and Good Clinical Practice guidelines were adhered to throughout the study.

### Antenatal and perinatal care

All participants received routine clinical care as per national guidelines. In the UK, this involved antenatal clinic visits every 2–4 weeks, with 4–6 visits including CGM and HbA_1c_ measurements. In Denmark, antenatal clinic visits occurred every 2 weeks, with five study visits at 8, 12, 21, 27 and 33 weeks gestation. These study visits included CGM and HbA_1c_ measurements. CGM profiles were collected over 5–7 days. Both studies used comparable glucose targets to achieve optimum glucose control; in the UK, these were: <5.5 mmol/l before meals, <7.8 mmol/l at 60 min postprandial and <6.7 mmol/l at 120 min postprandial. In Denmark, glucose targets were set at 4.0–6.0 mmol/l before meals, 4.0–8.0 mmol/l at 90 min postprandial and 6.0–8.0 mmol/l before bed.

### CGM

Continuous glucose monitors were used to record electrochemically measured subcutaneous interstitial glucose concentrations every 5 min, generating 288 measurements per day. Both studies used Medtronic CGM systems (MiniMed, Medtronic, Northridge, CA, USA), with CGM Gold sensors being used in the UK and Guardian REAL-Time CGM with Sof-sensors being used in Denmark. Monitors were calibrated against capillary blood glucose measurements as per the manufacturer’s instructions.

### HbA_1c_

Blood samples for HbA_1c_ measurements were obtained regularly throughout pregnancy at both centres. Samples were analysed locally by assays that were DCCT-aligned and from laboratories with National Glycohemoglobin Standardization Program (NGSP) certification.

### Statistical analysis

Average glucose was calculated as the mean of all glucose values obtained in the 5–7 day CGM profile. The corresponding week of gestation was noted for glucose values and, for analysis, values were paired with the HbA_1c_ values that had been measured within ± 1 week of the CGM profile. Each calculated average glucose value was matched to an individual HbA_1c_, though women contributed multiple average glucose–HbA_1c_ pairs across their pregnancy. A mixed-effects regression model was therefore used to account for the intra-individual variation with multiple data pairs per woman. In seeking the best-fitting model for the relationship between average glucose and HbA_1c_, this model included the covariates: gestational age in weeks, HbA_1c_ level and study centre (all centred to their grand mean). The models were explored for linear and curvilinear (squared) relationships, with model fit being assessed using the Akaike information criterion [27], whereby a lower score indicated a better fit of the model. All analyses were conducted in Stata 13, version 13 (StataCorp, College Station, TX, USA).

## Results

### Relationship between average glucose levels and HbA_1c_ in pregnancy

A total of 688 CGM profiles with a mean of 1275 (range 313–2839) glucose measures per profile were obtained for comparison with 688 HbA_1c_ levels. Each woman contributed an average of six average glucose–HbA_1c_ pairs across their pregnancy, at between 8 and 36 weeks gestation.

**Fig. 1:**
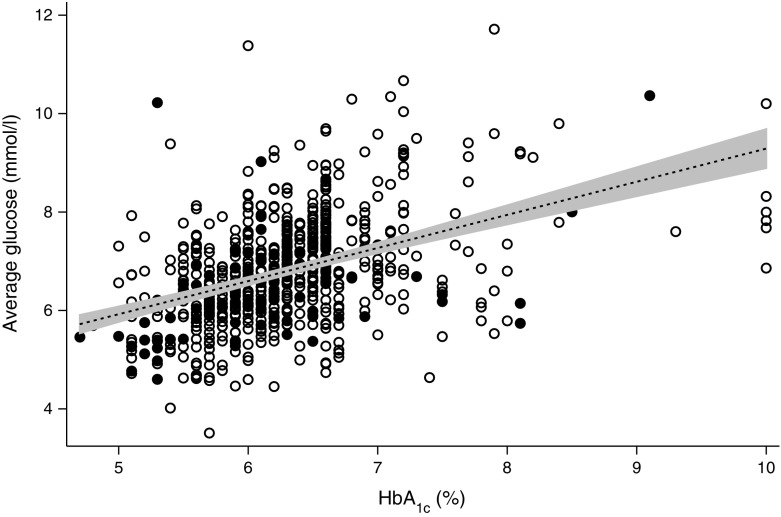
Average glucose against HbA_1c_ in diabetes. A graph showing average glucose vs HbA_1c_ with a linear fit and 95% CI. White circles, women with type 1 diabetes; black circles, women with type 2 diabetes. To convert values for HbA_1c_ in % into mmol/mol, subtract 2.15 and multiply by 10.929

Fig. 1 shows the association between average glucose and HbA_1c_ values obtained during pregnancy. A linear regression line, with 95% CI, is fitted to the data points (*r* ^2^ = 19.6%; average glucose–HbA_1c_ slope = 0.67 [0.57, 0.78]), showing a strong positive association. This implies that, for these women, on average a 1% (11 mmol/mol) difference in HbA_1c_ corresponded to a 0.67 mmol/l difference in their average glucose levels.

### Determining the best-fitting model to account for how gestational changes in HbA_1c_ influence the average glucose–HbA_1c_ relationship

An intercept-only mixed-effects model was compared with models containing random effects for the slope of the average glucose values in relation to HbA_1c_ levels (Table 1 and electronic supplementary material [ESM] Table 1). As the model containing the random effects of the slope coefficient provided a significant improvement in fit, the random slope was retained. The best-fitting model, with the lowest Akaike information criterion score, was model 5 (see Table 1). This model fitted average glucose to HbA_1c_, study centre, gestation in weeks (linear) and gestation in weeks squared (curved). Model 6 examined an interaction between HbA_1c_ and gestation in weeks to determine whether the gradient between average glucose and HbA_1c_ changed during pregnancy; the findings showed that it did not. There was also no interaction between HbA_1c_ and study centre, demonstrating that the relationship between average glucose and HbA_1c_ was consistent across the two datasets.

**Table 1:** Comparison of an intercept-only mixed-effects model with models containing random effects to determine the best-fitting model to account for how gestational changes in HbA_1c_ influence the average glucose–HbA_1c_ relationship

Model	AIC	Fixed effects	Intercept	HbA_1c_	Other covariates
1	1911.58	Intercept only	6.88 (6.70, 7.05)		
2	1762.65	+ HbA_1c_	6.84 (6.69, 7.00)	0.57 (0.37, 0.77)	
3	1759.12	+ HbA_1c_ + Centre	6.79 (6.63, 6.95)	0.55 (0.35, 0.75)	−0.39 (−0.70, −0.08)
4	1753.49	+ HbA_1c_ + Centre + Gestation	6.77 (6.61, 6.93)	0.43 (0.22, 0.64)	−0.43 (−0.75, −0.12) −0.01 (−0.02, −0.00)
5	1750.17	+ HbA_1c_ + Centre + Gestation + Gestation^2^	6.78 (6.62, 6.94)	0.50 (0.28, 0.72)	−0.39 (−0.70, −0.07) 0.04 (−0.01, 0.08) −0.001 (−0.002, −0.000)
6	1752.15	+ HbA_1c_ + Centre + Gestation + Gestation^2^ + HbA_1c_ × Gestation	6.78 (6.62, 6.94)	0.50 (0.28, 0.72)	−0.39 (−0.70, −0.07) 0.03 (−0.01, 0.08) −0.001 (−0.002, −0.000) 0.00 (−0.01, 0.02)

Data shown as regression coefficient (95% CI)

The mixed-effects models were fit between average glucose as the outcome and explanatory variables, using time nested within each mother

AIC, Akaike information criterion

### Deriving a pregnancy-specific eAG

**Fig. 2:**
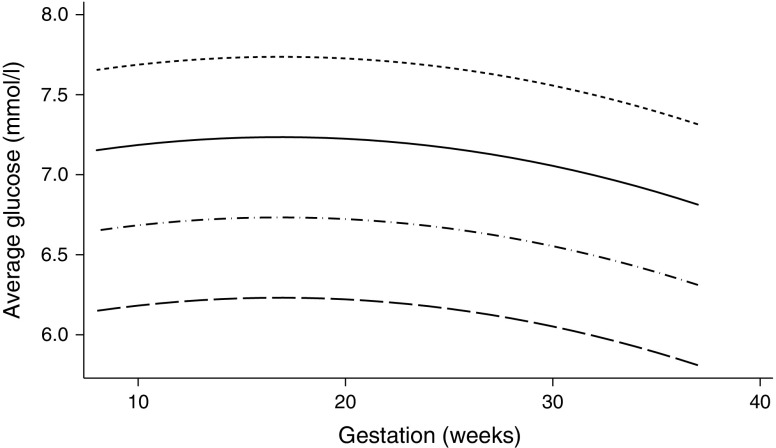
Changes in PeAG during gestation calculated for a range of HbA_1c_ using the best-fitting model. Long dash, 5.0% (31 mmol/mol) HbA_1c_; dash/dot, 6.0% (42 mmol/mol) HbA_1c_; solid line, 7.0% (53 mmol/mol) HbA_1c_; short dash, 8.0% (64 mmol/mol) HbA_1c_

Using the best-fitting curvilinear model, Fig. 2 shows the study mean pregnancy-specific eAG (PeAG) levels changing with gestational week for a range of HbA_1c_ levels. As an example, if the HbA_1c_ is measured at 6.0% (42 mmol/mol) during the 12th week of gestation, the PeAG is 6.7 mmol/l, whereas if it is measured at 36 weeks gestation the PeAG is 6.4 mmol/l. To estimate PeAG at any given week during pregnancy, the following equation can be used:

$$ \begin{array}{l}\mathrm{Glucose}\kern-0.15em \left(\mathrm{mmol}/\mathrm{l}\right)\kern-0.25em =\kern-0.15em 6.78\kern-0.35em +\kern-0.35em \left[0.43\times \left({\mathrm{HbA}}_{1\mathrm{c}}\ \left[\%\right]\kern0.3em -\kern0.3em 6.3\right)\right]\hfill \\ {}\kern9em +\kern-0.6em \left[0.04\times \left(\mathrm{Gestation}\ \left[\mathrm{weeks}\right]\kern0.3em -\kern0.3em 21\right)\right]\hfill \\ {}\kern9.3em  - \left[0.001\times \left(\mathrm{Gestation}\ {\left[\mathrm{weeks}\right]}^2\kern0.3em -\kern0.3em 528\right)\right]\hfill \end{array} $$

### Comparison of PeAG with the ADAG-calculated eAG

The eAG derived using the ADAG formula and that derived by our pregnancy-specific equation for a given value of HbA_1c_ are shown in Table 2. If we were to use the ADAG formula, an HbA_1c_ of 6.0% (42 mmol/mol) would equate to an eAG of 7 mmol/l irrespective of the gestational week, whereas it would equate to a lower PeAG (between 6.4 and 6.7 mmol/l depending on gestational week) using our pregnancy-specific equation. This difference is more pronounced at higher levels of HbA_1c_, where an HbA_1c_ of 8.0% (64 mmol/mol) equates to a PeAG of 7.7 mmol/l (at 12 weeks gestation and a PeAG of 7.4 mmol/l at 36 weeks gestation but, in contrast, using the ADAG formula, the same HbA_1c_ value would equate to an eAG of 10.2 mmol/l throughout gestation [8].

**Table 2:** Comparison of eAG values calculated from varying levels of HbA_1c_ using the ADAG calculation, vs the PeAG calculation.

HbA_1c_ % (mmol/mol)	ADAG eAG mmol/l	PeAG mmol/l		
		12 weeks gestation	24 weeks gestation	36 weeks gestation
5.0 (31)	5.4	6.2	6.2	5.9
6.0 (42)	7.0	6.7	6.7	6.4
7.0 (53)	8.6	7.2	7.2	6.9
8.0 (64)	10.2	7.7	7.7	7.4

## Discussion

This is the first study to examine the relationship between average glucose levels obtained by CGM and HbA_1c_ levels during pregnancy in women with diabetes. Our analysis demonstrates a positive linear relationship between average glucose and HbA_1c_ levels, but the slope is shallower than that reported in non-pregnant adults [8]. This validates the use of HbA_1c_ to represent average glucose levels during pregnancy, but suggests that a change in HbA_1c_ during pregnancy reflects a smaller change in average glucose than that assumed using the ADAG model [8, 12]. In addition, while we have shown that the relationship between average glucose and HbA_1c_ is stable during pregnancy, the absolute mean eAG varies with gestational week. Consequently, HbA_1c_ in pregnancy is associated with a lower eAG than that calculated by ADAG and this difference becomes more marked later in pregnancy. This means that the standard eAG reported with HbA_1c_ is not representative of average glucose levels in pregnancy and should not be used for assessing glucose control in pregnancy. We provide an alternative pregnancy-specific calculation for PeAG based on the observed relationship of HbA_1c_ and average glucose during pregnancy.

The work of the ADAG team has embedded the translation of eAG from HbA_1c_ into routine clinical practice [8, 12]. However, the ADAG analysis deliberately excluded pregnant women because of pregnancy-related physiological changes in HbA_1c_ [8] and, as a result, the routinely derived eAG may not be applicable to this population. HbA_1c_ is known to fall with the physiological changes associated with pregnancy, particularly in early and late pregnancy [13–17]. A strength of our study is that average glucose and HbA_1c_ data were obtained on repeated occasions (a mean of six times) in the same woman throughout pregnancy, enabling us to take account of gestational week in our data analysis. This revealed the stability of the average glucose–HbA_1c_ relationship across pregnancy.

While our data confirm that a positive association exists between average glucose and HbA_1c_, the slope of the relationship was shallower than that seen in non-pregnant adults [7–9], and this gradient remained stable during the last two trimesters of pregnancy. For example, previous data indicate that a 1% (11 mmol/mol) difference in HbA_1c_ is equivalent to a 1.0–2.0 mmol/l difference in average glucose [7–9, 11], whereas our data show that in pregnancy a much smaller difference in average glucose, 0.67 mmol/l, equates to a 1% (11 mmol/mol) difference in HbA_1c_. This suggests that a change in HbA_1c_ during pregnancy reflects a smaller change in average glucose compared with that seen outside of pregnancy.

Having established that the gradient between the average glucose–HbA_1c_ relationship is stable from the first trimester in pregnancy, there are nevertheless challenges when translating HbA_1c_ levels to eAG values, given the physiological changes that occur in pregnancy. The implication of a fall in HbA_1c_ or average glucose levels as pregnancy progresses means that any fluctuations in either become more sensitive to the gradient relationship. We have shown that gestational week is an important factor to account for when calculating an eAG from the average glucose–HbA_1c_ relationship during pregnancy, since mean levels of average glucose vary throughout pregnancy. Using our best-fitting model, the HbA_1c_ during pregnancy translates to a PeAG that is of a magnitude of 0.5–2.8 mmol/l difference compared with the eAG obtained using the ADAG formula that laboratories report [8, 12], and this difference is more pronounced at higher levels of HbA_1c_ (Table 2). This means that pregnant women and their clinicians could be misled by the standard eAG readings currently generated for laboratory reports and by automated online calculators. Furthermore, many glucose-monitoring devices generate an estimated HbA_1c_ from average glucose data. It is likely that this HbA_1c_ estimation is currently based on the ADAG formula, which may also be unintentionally misleading during pregnancy.

We consider that our analysis performed in pregnant women builds substantially on the ADAG team’s work. It is important, however, to note that while there are similarities, there are also several differences between our analysis and that of the ADAG. In contrast to the prospectively designed ADAG study [8], ours and other studies [9, 11] were pragmatic and made use of existing clinical data obtained from other studies. Compared with the ADAG study, which used 507 participants, of whom 427 had diabetes [8], our study is relatively small and we recognise that a larger study would help to improve the precision of our model to more confidently ascertain the relationship between average glucose and HbA_1c_ levels. In addition, the ADAG study included participants with a greater range of HbA_1c_ levels, including many with far higher HbA_1c_ values than the participants in our study.

The ADAG study paired the average glucose measures obtained by intermittent CGM readings taken for 2–3 days, every 4 weeks over a period of 3 months (giving ∼2500 glucose values per participant) to an HbA_1c_ taken at the end of the 3 months measurement period [8], resulting in one average glucose–HbA_1c_ pair per participant. In contrast, to address the complex issue of gestational physiological changes in HbA_1c_, the average glucose values obtained in our study were derived from an individual CGM session of 5–7 days, (giving a mean of 1275 glucose values), and were compared with an HbA_1c_ value taken within ± 1 week of the CGM profile, yielding a mean of six average glucose–HbA_1c_ pairs per participant. Previous small studies conducted in the 1980s used capillary blood glucose testing to calculate average glucose in pregnancy and showed a strong positive correlation between HbA_1c_ and the preceding 8–12 weeks’ average glucose values [28, 29]. We obtained the strongest relationship between average glucose and HbA_1c_ when both were measured within a few weeks of each other (ESM Table 2), suggesting that in pregnancy an HbA_1c_ value is more reflective of current average glucose readings (obtained by CGM) than those obtained over the preceding 3 months. Anecdotally, it is very common to see dramatic reductions in HbA_1c_ over very short periods of time (<4 weeks) at the start of pregnancy as women are motivated to rapidly optimise their glucose control upon finding out that they are pregnant; this may account for this more proximal relationship.

The ADAG study chose to weight their analysis with intermittent daytime capillary glucose readings but we did not. The rationale for weighting their analysis is unclear as: (1) CGM is already calibrated with regular capillary glucose readings; and (2) intermittent capillary glucose readings do not represent the ‘true’ average glucose value across the 24 h day since they are intermittent, ignore overnight glucose levels and may be skewed by postprandial glucose excursions. Since the ADAG analysis found that the average glucose–HbA_1c_ relationship was unchanged if only CGM readings were used for analysis, we decided to adopt this approach and not weight our analysis [8].

Our data have some further limitations; the women in our study were predominantly white European, which may limit applicability of our findings to women from other cultures and backgrounds. Our analysis did not include any women with gestational diabetes, so care needs to be taken with regard to its applicability in this context. We did not have data on haematocrit levels or iron deficiency/supplementation in our participants but, given that these are factors in the physiological changes of HbA_1c_ during pregnancy, this information might be useful to include in any future analysis of HbA_1c_ and average glucose levels in pregnancy.

We know from population-based studies that HbA_1c_ in pregnancy is a useful guide for pregnancy outcome and risk stratification [30] and is recommended by NICE for this purpose [20]. The ADA recommends regular assessment of glucose control during pregnancy, using monthly HbA_1c_, to maintain a level of 6.0–6.5% (42–48 mmol/mol) [22]; however, NICE was unable to make this recommendation because of a lack of data for validation of the relationship between HbA_1c_ to average glucose levels during pregnancy [20]. Our current analysis now provides this validation.

CGM is increasingly being used in clinical practice. The average glucose level calculated from the intensive longitudinal glucose data on these devices is far superior to that obtained by capillary glucose meters. Increasing the accessibility and use of CGM as an alternative to capillary glucose testing may significantly improve glucose management during pregnancy [24–26]. One of the difficulties of using CGM in pregnancy is determining exactly which aspects of glucose control to target. Targeting weekly PeAG could be a simple way to help women achieve the glucose control necessary to maintain their HbA_1c_ at ‘low risk’ levels across pregnancy. Our data would suggest that maintaining a PeAG of 6.4–6.7 mmol/l throughout pregnancy should achieve an HbA_1c_ of 6.0% (42 mmol/mol), which is necessary for reducing the risk of adverse pregnancy outcomes.

In summary, HbA_1c_ can be translated to eAG values in pregnant women with diabetes, but these are not the same as those commonly reported. Therefore, pregnancy-specific values, PeAG, are recommended for use in antenatal clinical care.

## Electronic supplementary material

Below is the link to the electronic supplementary material.

ESM Tables (PDF 160 kb)

## Abbreviations

ADAG: A1C-Derived Average Glucose study
CGM: Continuous glucose monitoring
eAG: Estimated average glucose
NICE: National Institute for Health and Care Excellence
PeAG: Pregnancy-specific estimated average glucose
