# Supplementary material for: Translating HbA1c measurements into estimated average glucose values in pregnant women with diabetes
Source: Diabetologia. 2017 Jan 19;60(4):618–24. doi: 10.1007/s00125-017-4205-7 (PMC6518090; doi:10.1007/s00125-017-4205-7)
Supplement: Supplementary file 1 — (PDF 160 kb) [file 125_2017_4205_MOESM1_ESM.pdf]

Translating HbA1c measurements into estimated average glucose values in pregnant women with diabetes. Electronic Supplementary Material

ESM Table S1: Fixed and random effects of best fitting model (model 5)

	Estimate (95% CI)
Fixed effects	
Intercept	6.78 (6.62;6.94)
HbA1c	0.50 (0.28;0.72)
Centre	-0.39 (-0.70;-0.07)
Gestation	0.04 (-0.01;0.08)
Gestation ^{2}	-0.001 (-0.002;-0.000)
Random effects	
var(hba1cCen)	0.40 (0.22;0.71)
var(constant)	0.45 (0.30;0.68)
var(Residual)	0.51 (0.45;0.57)

ESM Table S2: Linear regression between the average glucose and HbA1c measured in different trimesters.

	HbA1c		
Average glucose predicts	Trimester 1	Trimester 2	Trimester 3
Trimester 1	0.20	0.14	0.01
Trimester 2		0.13	0.05
Trimester 3			0.11

The r^2 was used to compare the model fit, with the greater r^2 indicating better model fit. As greater time elapsed between average glucose and HbA1c measurements the model fit worsened e.g. the average glucose taken in trimester 1 predicts the HbA1c in Trimester 1 better than that in Trimester2 or Trimester 3.
